# Prevalence of and factors associated with utilization of herbal medicines among outpatients in primary health centers in Cambodia

**DOI:** 10.1186/s12906-018-2181-1

**Published:** 2018-04-02

**Authors:** Hattie Pearson, Tyler Fleming, Pheak Chhoun, Sovannary Tuot, Carinne Brody, Siyan Yi

**Affiliations:** 1 College of Osteopathic Medicine, Touro University California, Vallejo, CA USA; 2 Public Health Program, Touro University California, Vallejo, CA USA; 3 KHANA Center for Population Health Research, Phnom Penh, Cambodia

**Keywords:** Herbal medicines, Traditional medicines, TCAM, Outpatients, Primary healthcare, Cambodia

## Abstract

**Background:**

Traditional, complementary and alternative medicine (TCAM) is seen as a way to provide healthcare in both developed and developing countries across the world. In Cambodia, there is a long tradition of using TCAM. However, scant studies have been conducted on the extent of Cambodian TCAM use and how it interacts with allopathic health care to date. In this study, we examined the prevalence of and factors associated with utilization of herbal medicines among patients with chronic diseases in primary health care settings in Cambodia.

**Methods:**

A cross-sectional survey was conducted in 2015 with outpatients receiving treatment and care for chronic diseases in two urban and two rural primary health centers purposively selected from Phnom Penh, Kampong Cham and Siem Reap. Every eligible patient was randomly selected at the health centers using a systematic sampling procedure. I-CAM-Q was used to measure TCAM use. A multivariate logistic regression model was constructed to identify factors associated with herbal medicine use.

**Results:**

In total, 1602 patients were included in this study, of whom 77.7% were female, and 51.2% were recruited from urban primary health centers with a mean age of 46.5 years (SD = 15.2). Of total, 27.0% reported at least one consultation with a TCAM provider in the past 12 months. The most common modality of TCAM used was herbal medicine (89%). Herbs were obtained at drug or folk stores (36.9%), from herbalists directly (28.5%) or from their own gardens (18.6%). Of herb users, 55.2% reported that herbs were somewhat helpful. After adjustment, herb users were significantly more likely to be female (AOR = 1.42, 95% CI = 1.12–2.67), have completed less schooling (AOR = 0.66, 95% CI = 0.45–0.96), were unemployed or homemakers (AOR = 0.23, 95% CI = 0.13–0.52) and have a gastrointestinal illness (AOR = 0.49, 95% CI = 0.39–0.62).

**Conclusions:**

Herbal medicines are broadly used among chronic disease patients in Cambodia. Understanding TCAM use in the general population will support health care practitioners and policy makers to make informed decisions about the use of TCAM. Integration of TCAM into the primary health system should be further explored.

## Background

Traditional, complementary and alternative medicine (TCAM) has been a growing area of interest in recent years. The World Health Organization (WHO) has made research and integration of TCAM as one of its global priorities in the upcoming decade [1]. In the WHO’s 2012 survey, countries around the world cited a paucity of adequate research as the most limiting factor in improving and integrating TCAM into their national healthcare system [1]. Reflecting similar trends, the Association of Southeast Asian Nations (ASEAN) has made it a priority to understand the uses and practices of TCAM in the region for better safety and economic regulation purposes [2].

A recent study of TCAM in the lower Mekong countries including Cambodia, Thailand and Vietnam found similar rates of use in each country with 76.7% of people reporting using some form of TCAM or seeing a TCAM provider in the past year [3]. Meanwhile, other studies have indicated high rates of use in Malaysia (63.9%) and Thailand (52.5%) [4]. These findings corroborate the results of a recent systematic review of TCAM literature, which found that the highest rates of TCAM use were in East Asian countries [5]. However, the popularity of TCAM is not limited to only East Asian countries. In a recent study of Cambodian refugees living in Long Beach, California, 34% of the respondents reported continued seeking of TCAM in addition to seeking western allopathic medical therapies [6], while the systematic review noted that, in the United Kingdom, United States and Canada, TCAM use in the general population ranged from 26% to 52% [5].

Cambodia offers a unique opportunity to examine the sociocultural, economic and medical reasons in patients who utilize TCAM. Between 1975 and 1979, the Khmer Rouge killed or detained physicians and closed the Cambodia’s only medical school; by 1979, only 45 western-medicine-trained physicians remained in the country [7]. As a result, Cambodia has one of the lowest physician-to-patient rates in the world [8]. Such malevolent history has combined with long-standing cultural acceptance and poor access to western allopathic therapies resulting in a high prevalence of TCAM use [7, 9, 10].

In Cambodia, out of pocket payment for medical services accounts for more than 60% of the total health expenditure, and can be a serious financial burden, particularly in poorer populations [11]. As herbal medicines are easily accessible within communities and traditional drug stores across the country, they have played an important role in Cambodian healthcare. According to the National Center of Traditional Medicine, approximately 40 to 50% of the population in remote areas use traditional medicine [12], and is often the most preferred healthcare option in Cambodia due to its accessibility, cultural history and limited access to more expensive modern healthcare services [12].

Cambodian traditional medicine is a combination of three explanatory models of disease: supernatural theory (magic), naturalistic theory (air, water) and maintenance of hot/cold balance [3, 7]. TCAM is often administered by elders called *kru khmer*, mediums known as *kru chol ruup* and Buddhist monks [10]. Although TCAM has not been officially integrated into the primary health care system, its importance has been strongly recognized by the Cambodian government. The Royal Government of Cambodia first formally encouraged the preservation and continuation of the existing TCAM practices in 1998 [12]. The Cambodian Ministry of Health established the National Center of Traditional Medicine in the early 2000s. In 2004, the country’s Prime Minister also declared that, “the Royal Government will continue to encourage the use of TCAM with appropriate information and control in conjunction with the use of modern medicine” [12].

Many of the studies about TCAM have sought to elicit the demographic characteristics and motivations why patients seek TCAM; yet consistency across the literature is lacking. Most studies support the increased use among females and people with higher education and reported poor health outcomes or perceived poor health among users in high-income countries [4, 13]; while in low- and middle-income countries, TCAM use has been associated with lower educational and socio-economic status [14–18]. Other areas of consensus in the literature include a significant association between TCAM use and the ‘chronic disease triad of arthritis, musculoskeletal disorders and stroke [15, 19–22] and depression [20, 23, 24]. A final common theme is patients not telling their primary care providers about TCAM use, with studies reporting ranges of 55 to 70% of patients using TCAM do not tell their primary care providers about the use [25–27].

Our study examined the prevalence of and factors associated with TCAM use among patients with chronic diseases in primary healthcare settings in Cambodia. To gain a clearer picture of typical Cambodian TCAM users, this paper focused on the use of herbs as the TCAM modality. Utilization of herbal medicines is frequently cited as one of the leading types of TCAM used by patients in the literature [3, 28, 29], and a previous study of TCAM found a high prevalence of herbal medicine usage in Cambodia [3]. Likewise, there have been numerous studies in resource poor settings that have specifically investigated herbal medicine usage [16–18]. Moreover we did inquire about a wider range of TCAM modalities including acupuncture, massage and other self help practices, but respondents rarely reported using any modality except herbal medicines, so we restricted our analyses to herbal medicine use.

## Methods

### Study sites and participants

A cross-sectional survey was conducted in 2015 as part of a multi-country study of ambulatory patients receiving treatment and care for chronic diseases in two urban (one in the capital city of Phnom Penh and another one in Kampong Cham provincial town) and two rural primary health centers (one in Kampong Cham and another one in Siem Reap). The primary health care centers are public facilities that provide a range of services including initial consultations and primary diagnosis, emergency first aid, chronic disease care, maternal and child care, birth spacing advice, immunization, health education and referral. Approximately 400 patients were randomly selected from each health center using a systematic sampling procedure. Sixteen patients refused the participation because of their time constraints and were replaced by the next eligible patient on a patient list provided by the participating health centers. A total of 1602 patients completed the interview in this study. Further details of the larger study are published elsewhere [3, 28].

A patient would be invited to participate in the study if he/she was: (1) aged older then 21 years; (2) able to communicate in Khmer; (3) willing to participate in the study and could provide a verbal informed consent; (4) able to present themselves on the day of the interview; (5) physically and mentally stable to participate in the study and (6) receiving treatment and care at the selected health centers for at least one of 20 chronic conditions including asthma, chronic obstructive pulmonary disease, diabetes mellitus, hypertension, dyslipidemia, coronary artery disease, cardiac failure, cardiac arrhythmia, stroke, arthritis, gout and other musculoskeletal conditions, epilepsy, Parkinson’s disease, liver disease, kidney disease, thyroid diseases and mental disorders [23, 29].

### Training and data collection

Data collection was conducted in August and September 2015 by interviewers who had experience in data collection under supervision of researchers from KHANA Center for Population Health Research. One interviewer was dispatched to each health center during the study period. All interviewers and field supervisors were trained for two days on data collection methods and one day for tool pretesting and reflection. The main objective of the training was to make sure that all interviewers and supervisors understood the data collection procedures and follow the standardized guidelines in the same manner to ensure the quality of the data. The training covered necessary skills including interview techniques, confidentiality and privacy as well as practices of the questionnaire administration. We also reviewed the study protocol during the training sessions in order for the team members to be thoroughly familiar with it.

The training also included quality control skills such as rechecking and reviewing the questionnaires after administration as well as resolving issues that might arise during the fieldwork. Regular review sessions with interviewers were conducted during the survey period to review progress and communicate any problems or issues occurring during the data collection. The estimated time for each interview, including time for obtaining informed consent, was approximately 30 min.

### Variables and measurements

The survey questionnaire was developed in English and then translated into Khmer, the national language of Cambodia, by the research team. Another researcher who was not involved in the initial questionnaire development back-translated it into English. We adapted the international tool to measure TCAM use (I-CAM-Q), which contains three sections [30]. Section 1 asks about “visiting health center providers”, section 2 about the “use of herbal medicine and dietary supplements” and section 3 about “self-help practices.” The treatment modalities are presented in the form of a list, and respondents provided information on their usage over the previous 12 months (yes/no) [30, 31]. The questionnaire inquired about a range of TCAM modality utilization including: homeopathy, vitamins/minerals, ginseng and various self-help practices including medication, yoga, Qigong, Tai Chi, relaxation and prayer. However, many of these modalities had less than 10% of respondents who reported using them and therefore, were not included in further analyses. Given that nearly half of the respondents (44.5%) had reported herbal medicine use, we focused our analyses on this particular modality of TCAM to avoid confounding the results of the study.

Other measures that were collected in the study included alcohol use assessed with the Alcohol Use Disorder Identification Test (AUDIT-C) [32] and tobacco use assessed using four items from the World Health Organization STEPS instrument [33]. The General Physical Activity Questionnaire was used to measure participant’s physical activity, type, frequency and food habits [33]. The questionnaire also collected information on chronic disease related stigma measured via the Chronic Illness Anticipated Stigma Scale (CIASS) [34] and adherence to medication assessed with the 8-item Morisky Medication Adherence Scale (MMAS) [35].

### Data management and analyses

Double data entry was performed to minimize errors using EpiData version 3 (Odense, Denmark). Herb users were defined as those who answered yes to the question if they have used herbal products in the last 12 months, while non-herb users were defined as people who answered no to the question if they had used herbal products in the last 12 months. We then compared herb users and non-herb users on demographics, medical conditions and health behaviors using chi-square test (or Fisher’s exact test when a cell count was smaller than five). A multivariate logistic regression model was constructed to identify factors associated with herb use based on the results of the initial bivariate analyses. Variables that were found to be significantly associated with herb use were pushed through the logistic regression. Adjusted odds ratio (AOR) were obtained and presented with 95% confidence intervals (CI) and *p*-values. Two-sided *p*-values < 0.05 was used to indicate statistical significance. STATA version 14 (StataCorp, LP, Texas, USA) was used for all data analyses.

## Results

### Demographics and medical diagnoses

The demographics of our sample population are displayed in Table 1. The population was mostly female (77.6%), currently married (77.3%) and living in a rural location (63.5%). The vast majority reported a Buddhist religion (96.8%) and spoke Khmer as their primary language (98.3%). Almost 70% had some formal education, with 55.7% of the sample attaining at most a primary school education. Over half reported being unemployed or staying at home as homemakers for their family (55.0%). Upon comparison, a significantly higher proportion of herb users were female (*p =* 0.004), had less formal schooling (*p* < 0.001) and were homemakers or unemployed (*p =* 0.001) compared to non-herb-users. The most common ailments were gastrointestinal in nature with 71.9% of the surveyed group reporting having been treated for a gastrointestinal illness in the last 12 months. Upon comparison, most herb users and non-users had similar rates of medical illnesses; herb users were more likely to report having been treated for a gastrointestinal illness in the last 12 months (*p <* 0.001). Herbs were also utilized significantly more by those with hypertension as compared to those with other diseases or conditions (*p =* 0.01).

**Table 1:** Comparison of socio-demographic characteristics among herb users and non-herb-users

Socio-demographic characteristics	Total	Herb users	Non-herb-users	
	(*n* = 1592)	(*n* = 708)	(*n* = 884)	
	*n* (%)	*n* (%)	*n* (%)	*p*-value^*^
Age				0.51
21–30	301 (18.9)	127 (17.9)	174 (19.7)	
31–40	308 (19.4)	150 (21.2)	158 (17.9)	
41–50	291 (18.3)	123 (17.4)	168 (19.0)	
51–60	390 (24.5)	180 (25.4)	210 (23.7)	
61–70	218 (13.7)	95 (13.4)	123 (13.9)	
> 70	84 (5.2)	33 (4.7)	51 (5.8)	
Gender				0.004
Male	359 (22.4)	134 (18.9)	220 (24.9)	
Female	1243 (77.6)	574 (81.1)	664 (75.1)	
Marital Status				0.42
Never married	130 (8.1)	52 (7.3)	76 (8.6)	
Currently married	1238 (77.3)	559 (78.9)	674 (76.2)	
Divorced/separated	234 (14.6)	97 (13.7)	134 (15.2)	
Education				0.001
No schooling	492 (30.7)	232 (32.7)	258 (29.2)	
Primary school	892 (55.7)	405 (57.2)	481 (54.4)	
Secondary school	162 (10.1)	60 (8.5)	101 (11.4)	
University or higher	56 (3.5)	11 (1.6)	44 (5.0)	
Location				0.67
Urban	585 (36.5)	264 (37.3)	321 (36.3)	
Rural	1017 (63.5)	444 (62.7)	563 (63.7)	
Occupation				< 0.001
Homemaker	766 (55.1)	367 (60.3)	399 (51.2)	
Unemployed	15 (1.1)	2 (0.7)	13 (1.7)	
Retired/disabled	43 (3.1)	18 (3.0)	25 (3.2)	
Student	54 (3.9)	11 (1.8)	43 (5.5)	
Self-employed	421 (30.3)	171 (28.1)	250 (32.0)	
Employed	90 (6.5)	40 (6.5)	50 (6.4)	
Religion				0.99
Buddhist	1550 (96.8)	685 (96.8)	855 (96.7)	
Muslim	34 (2.1)	15 (2.1)	19 (2.2)	
Christian	18 (1.1)	8 (1.1)	10 (1.4)	
Medical conditions				
Respiratory conditions	223 (14.2)	101 (6.4)	102 (6.5)	0.81
Cardiac conditions	285 (18.2)	133(8.5)	152(9.7)	0.44
Neurological conditions	220 (13.8)	95(6.0)	125(7.9)	0.68
Musculoskeletal conditions	173 (11.0)	86 (12.3)	85 (9.8)	0.11
Gastrointestinal	1149 (71.9)	562 (79.4)	580 (65.8)	< 0.001
Hypertension	403 (25.2)	156 (22.3)	244 (27.8)	0.01
Diabetes	184 (11.5)	86 (12.2)	97 (11.0)	0.48
Psychiatric	74 (4.7)	32 (4.6)	42 (4.9)	0.79

*Chi-square test or Fisher’s exact test (when a cell count was smaller than 5) was used

### Health behaviors

Table 2 summarizes a range of health habits, practices and beliefs between herb users and non-herb-users. There were no statistically significant differences between the two groups in regards to tobacco or alcohol use or self-help practices. Overall, the majority of respondents did not use tobacco (89.7%) or alcohol (74.8%), and did not exercise in the last week (64.5%). When asked to assess their quality of life, slightly less than half of the participants rated their quality of life as good/very good (46.2%). People who reported not using herbs were more likely to report a good/very good quality of life, while people who reported using herbs were more likely to report a neither good nor poor quality of life (*p* < 0.001).

**Table 2:** Comparison of health behaviors among herb users and non-herb-users

Health behaviors	Total	Herb users	Non-herb-users	
	(*n* = 1592)	(*n* = 708)	(*n* = 884)	
	*n* (%)	*n* (%)	*n* (%)	*p*-value^*^
Current cigarette smokers	164 (10.3)	69 (9.7)	95 (10.7)	0.52
Alcohol use				0.2
Never	1197 (75.2)	538 (76.1)	659 (74.5)	
Monthly or less	278 (17.5)	112 (15.8)	166 (18.8)	
2–4 times a month	83 (5.2)	45 (6.4)	38 (4.3)	
2–3 times a week	29 (1.8)	10 (1.4)	19 (2.1)	
4 or more times a week	4 (0.3)	2 (0.3)	2 (0.2)	0.17
Exercised during the past week	563 (35.5)	225 (33.3)	308 (34.4)	0.59
Self-help utilization	765 (48.1)	352 (49.7)	413 (46.7)	0.23
Quality of life				< 0.001
Poor/very poor	204 (12.9)	82 (11.7)	122 (13.9)	
Neither poor nor good	645 (40.9)	326 (46.6)	320 (36.5)	
Good/very good	727 (46.2)	292 (41.7)	435 (49.6)	
Type of treatment sought for a chronic condition				
Herbalist	325 (18.3)	303 (31.7)	19 (2.3)	< 0.001
Medical	1449 (81.7)	653 (68.3)	790 (97.7)	0.04
Would a healthcare worker blame you for your medical condition?				0.08
Very unlikely	1158 (73.1)	531 (75.6)	627 (69.8)	
Unlikely	360 (22.7)	141 (20.1)	219 (24.5)	
Neither	30 (1.9)	16 (2.3)	16 (1.8)	
Likely	34 (2.1)	14 (2.0)	20 (2.3)	
Very likely	2 (0.1)	0 (0.0)	2 (0.2)	
Type of condition addresses with herbal remedies [Herbal users only]				
Acute	170 (24.3)			
Chronic	394 (56.9)			
Improvement of overall health	129 (18.6)			

^*^Chi-square test or Fisher’s exact test (when a cell count was smaller than 5) was used

Approximately one-fifth (18.3%) had visited an herbalist in the last 12 months. Nearly one-half (43.7%) of herb users had visited an herbalist in the last 12 months, and herb users were significantly more likely to have visited an herbalist than non-herb-users (*p <* 0.001).

Lastly, we explored perceived attitudes of medical providers towards survey respondents between herb users and non-herb-users. The majority of respondents (73.1%) reported it was very unlikely that a medical provider would blame them for their medical conditions. There was no significant difference between herb users and non-herb-users for perceived attitudes of healthcare providers.

### Types of conditions and level of satisfaction with herbal remedies

Among participants that indicated using an herbal medicine in the last 12 months, one-fourth (24.3%) used herbs for acute problems, while over half (56.9%) used herbs for chronic problems. A small proportion (18.6%) used herbs to improve their overall health. Out of respondents who reported using herbs in the last 12 months, approximately one-third (37.4%) reported the herbs were “very helpful,” while over half (56.8%) reported the herbs were “somewhat helpful.”

### Factors associated with utilization of herbal medicines

Table 3 shows the results of a multivariate logistic regression analysis exploring factors associated with herb use. After controlling for other covariates in the model, participants with secondary school education (AOR = 0.64, 95% CI = 0.44–0.94) and university or higher education (AOR = 0.27, 95% CI = 0.13–0.55) were significantly less likely to be herb users compared to those who had no formal education. Participants who reported having been treated for hypertension (AOR = 1.29, 95% CI = 1.02–1.64) and gastrointestinal diseases (AOR = 1.99, 95% CI = 1.57–2.53) were significantly more likely to be herb users than those who had no such conditions. Regarding quality of life, participants who perceived their quality of life as ‘neither poor nor good’ were significantly more likely to be herb users compared to those who perceived their quality of life as ‘poor/very poor’ (AOR = 1.56, 95% CI = 1.12–2.16).

**Table 3:** Factors associated with herb use among patients with chronic diseases

	AOR (95% CI)	*p*-value^*^
Gender		
Male	Reference	
Female	1.24 (0.96,1.60)	0.10
Level of formal education		
No education	Reference	
Primary school	0.91 (0.72, 1.15)	0.42
Secondary school	0.64 (0.44, 0.94)	0.02
University or higher	0.27 (0.13, 0.55)	< 0.001
Hypertension		
No	Reference	
Yes	1.29 (1.02, 1.64)	0.04
Gastrointestinal diseases		
No	Reference	
Yes	1.99 (1.57, 2.53)	< 0.001
Quality of life		
Poor/very poor	Reference	
Neither poor nor good	1.56 (1.12, 2.16)	0.008
Good/very good	1.06 (0.77, 1.47)	0.72

Abbreviations: AOR, adjusted odds ratio; CI, confidence interval

^*^Variables significantly associated with herb use in bivariate analyses were simultaneously included in a multivariate logistic regression model

## Discussion

This study revealed some interesting trends, some of which are prevalent in the literature and some that go against the prevailing trends of TCAM use. One major finding of our work is that, while being of female gender was associated with likelihood utilization of herbal medicines in bivariate analyses, the association did not withstand the logistic regression when other variables were controlled. An overwhelming number of the studies conducted both in Asian and Western countries have found that being female significantly increases the likelihood of utilization of herbal medicines [3, 5, 20, 28, 29, 36]. This finding is supported by some other research, specifically a Thai study that assessed the prevalence of herbal supplements in patients receiving ambulatory chronic kidney disease services. Like our study, it was a cross-sectional survey, and the only demographic difference found between herb users and non-herb-users was that non-herb-users were more likely to be smokers, but gender was not a significant factor [26]. Other research has shown that, in other resource-poor settings such as Africa, female gender has not been associated with TCAM usage [16, 18]. Another study conducted in Cambodia exploring the use of TCAM had found that females were more likely to use TCAM, specifically self-help practices [3]. In the previous study, only two locations were surveyed and the populations reported an overall higher level of education [3], therefore the discrepancies between the study samples could be reflected by the unique characteristics of the populations surveyed.

One of the other findings of our study was that herbal medicine usage was associated with lower educational levels. Like female gender, most studies in more developed countries have found higher educational attainment was more associated with TCAM use [4, 5, 13, 37]. However, in resource-poor settings, TCAM usage has generally been associated with lower level of educational attainment [14, 15, 17, 18]. Given Cambodia’s tragic history, access to allopathic medicine ranks among some of the lowest in the world. The 2010 World Bank’s statistics give Cambodia a doctor-to-1000-population ratio of 0.21, much lower than that in neighboring Vietnam (1.11), and still lower than that in Myanmar (0.50) and Laos (0.27) [8]. Therefore, it makes some sense that the picture of herbal medicine usage in Cambodia aligns more with other resource-poor countries such as those in sub-Saharan Africa where doctors-to-population ratios are similar (0.01–0.37) [8].

Like previously published literature, our work demonstrated that herbal medicine usage was associated with several different medical conditions, in particular a history of hypertension and gastrointestinal illnesses. Unfortunately, it is difficult to compare this finding to a majority of the studies as most use a much wider definition of TCAM; and as already described, have found that conditions namely the ‘chronic disease triad of arthritis, musculoskeletal disorders, and stroke’ and depression were key diagnoses related to TCAM use without making mention of hypertension and gastrointestinal diseases [15, 19–24]. Our findings maybe attributable to our specific focus on herbal remedies, which maybe more commonly used for these diagnoses, while other modalities like acupuncture, message, etc. may be used for other diagnoses like arthritis or depression. However, the reported usage of these modalities in our population was too low for analyses.

Our final finding is well corroborated by the literature and that is an association between an ambivalent quality of life (defined in our study as response of neither poor nor good in the quality of life section of the survey) and an increase utilization of herbal remedies. Other studies have slightly varied methods of asking a similar question including ‘more unhealthy days,’ ‘perceived poor health’ or self-reported ‘poorer health status,’ but they have found that patients who are sicker, or feel sicker do use TCAM more often [6, 15, 21, 38–43]. There may be many reasons for this finding including the need of frequent treatment or desiring more constant symptomatic palliation in chronic conditions, the greater availability of herbal remedies, which maybe attained at a lower cost, can be made in the home or are deeply embedded cultural ‘home remedies.’ Further work on this topic could be to try and delineate the roles of income or resource availability in this relationship and if those factors affect whether patients choose TCAM or more frequent allopathic medical remedies.

Our analysis did not look into the harmful effects of many herbs; however, previous studies have catalogued a number of adverse events associated with herbal medicine usage including Stevens Johnson’s syndrome, poisoning, anemia, thrombocytopenia and teratogenicity [17, 44, 45]. Given the different remedies used among different cultures and the unique properties of each plant, it is impossible to ascertain the safety of herbal medications without knowing the botanical ingredients. However, further research into specific formula and usages in Cambodia would help ascertain the safety of these medications.

There are several limitations in this study. First, this was a cross-sectional study and therefore, we are unable to draw causal inferences from our findings. Second, all measures were self-reported which relied on a patient’s recall ability and may have lead to both under and over-reporting of certain variables, although validated measures were used to address this problem. Another limitation is one already documented in the literature on the topic of TCAM is the difficulty of external validity [20]. Different studies have varied definitions of TCAM and different countries have varied levels of TCAM integration into their health systems. Therefore, some modalities of TCAM are seen in one culture and not in others. Additionally, data are often gathered through interviews where translators may have different understandings of terminology. While our study focused on herbal medicine due to a high incidence of reported usage among our survey respondents, many other studies have focused on a more general definition of TCAM. Likely a larger sample size would be needed to adequately study the other modalities in which we found lower levels of usage such as yoga, massage and self-help practices. However, other studies that have looked a specifically herbal medicine usage in low- and middle-income countries have had similar results to ours [16–18]. Additionally, our recruitment at primary health centers could have skewed our results as some patients may be exclusively using TCAM for their health care needs and may not visit a health facility.

## Conclusions

Our study found that herbal medicines are broadly used among patients with chronic diseases in Cambodia with nearly half of the population having used an herbal medicine in the last 12 months. Interestingly, there were little differences based on age, gender or geographical living locations, indicating that this practice is popular with a broad segment of the population. People who were unemployed were more likely to be using herbs; however, we are unable to tell whether they are using herbs due to some illnesses or injuries that prevent them from working or some other factors. Though we did not directly measure socioeconomic status of our study population, participants who had obtained a higher level of education were less likely to use herbal medicines. People with gastrointestinal conditions and hypertension were significantly more likely to report using herbs. High levels of satisfaction with use were seen among those who used herbs for chronic conditions. More research would be needed to determine the types of herbs used, conditions treated, desired effects and a better detail of the factors and their interaction that motivate herb use. Future studies quantifying and describing other TCAM modalities found in the literature such as acupuncture, cupping, meditation, or prayer across a wider geographic swath of Cambodian geography would also contribute to the growing portrait of herb and TCAM use in Cambodia. Since the importance of TCAM is well accepted by the population and officially supported by the Cambodian government [10, 12], integration of TCAM into the primary health care system should be further encouraged and supported by more specific research.

## Abbreviations

AOR: Adjusted odds ratio
ASEAN: Association of Southeast Asian Nations
AUDIT-C: Alcohol Use Disorder Identification Test
CI: Confidence interval
CIASS: Chronic Illness Anticipated Stigma Scale
HIV: Human immunodeficiency virus
MMAS: Morisky Medication Adherence Scale
NECHR: National Ethics Committee for Health Research
TCAM: Traditional, complementary and alternative medicine
WHO: World Health Organization
